# Lifespan of COVID‐19 living guideline recommendations: A survival analysis

**DOI:** 10.1002/gin2.12012

**Published:** 2024-08-31

**Authors:** Emma McFarlane, Toby Mercer, Steve Sharp, Debra Hunter, Kate Kelley, Fiona Glen, Maria Majeed

**Affiliations:** ^1^ National Institute for Health and Care Excellence Manchester UK

**Keywords:** COVID‐19, lifespan of recommendations, living guidelines, updating guidelines

## Abstract

**Background:**

NICE has maintained a portfolio of coronavirus disease 2019 (COVID‐19) living guidelines since March 2020. Recommendations within these living guidelines are subject to continuous surveillance and updates in response to triggers. However, the lifespan of individual living guideline recommendations and features that may impact whether a recommendation becomes out of date sooner is unknown.

**Objectives:**

This study describes the length of time NICE COVID‐19 living guideline recommendations have remained valid.

**Methods:**

All guidelines within NICE's COVID‐19 portfolio were included to determine the lifespan of living guideline recommendations. Data were collected on all recommendations that had been developed, undergone surveillance or updated between 1 March 2020 and 31 August 2022. The initial publication date, decision to update and updated publication date were extracted. Updates were labelled as major changes in evidence synthesis or minor changes without a substantial change in the evidence base. Any recommendation that had not been updated or withdrawn was censored. Survival analysis (Kaplan–Meier curve) was carried out to determine the lifespan of recommendations.

**Results:**

Overall, 26 COVID‐19 living guidelines and 1182 recommendations were included in the analysis. Living recommendations had a median survival time of 739 days (interquartile range [IQR]: 332, 781). Based on recommendation type, intervention recommendations had a shorter survival time (354 days, IQR: 312, 775) compared to diagnosis (368 days, IQR: 328, 795), patient experience (733 days, IQR: 345, 795) and service delivery (739 days, IQR: 643, 781). Within intervention type, pharmacological recommendations had the shortest survival time versus nonpharmacological recommendations (335 days, IQR: 161, 775 vs. 775 days, IQR: 354, 775). Updates were published an average of 29.12 days following a surveillance decision.

**Conclusion:**

Within living guidelines, some recommendations need to be updated sooner than others. This study outlines the value of a flexible responsive approach to surveillance according to the pace of change and expectation of update triggers.

## Key points

Key findings

- Within the context of a living guideline, some recommendations will become out of date sooner than others.

What this adds to what was known?

- This study supports the concept of prioritising recommendations within a guideline to be living.

What is the implication and what should change now?

- Guideline developers should consider which recommendations within a living guideline would have the most value in being maintained as living to optimise resources.

## 1 INTRODUCTION

Living guidelines provide continually updated advice based on the best available evidence.^1^ Living guidelines are considered the most useful in high‐priority clinical areas where there is uncertainty and a high likelihood of emerging new evidence.^2^ This approach has been useful in the context of the coronavirus disease 2019 (COVID‐19) pandemic as creating living guidelines on managing COVID‐19 has enabled guideline developers to update recommendations quickly in response to dynamic changes in the evidence base.

To achieve living guidelines, surveillance is conducted at frequent intervals and evidence or system intelligence is rapidly considered for its impact on recommendations.^3^ Updates to recommendations are undertaken as soon as possible after a trigger indicates the need to update a guideline. Updates to recommendations may be in response to several triggers, which are events that may impact guideline recommendations at any time after guideline publication and could include^4^:

- publication of a study that is directly relevant to the guidance and has the potential to affect existing recommendations
- substantial changes in policy or legislation (an example includes changes to the UK physical activity guidelines by the Chief Medical Office)
- withdrawal of a drug from the market, or a clinically significant drug safety update from the Medicines and Healthcare products Regulatory Authority or the Commission on Human Medicines
- real‐world data indicating a change in the disease that significantly changes the hospitalisation or mortality rate.

In a living guideline, the unit of update is generally individual recommendations, meaning these can be updated as relevant new evidence emerges, without the need to wait for the entire guideline to be revised.^5^

A previous survival analysis of a cohort of published NICE clinical guidelines found that the median lifespan of guidelines was 60 months.^6^ Although it is recognised that individual recommendations within a living guideline will be faster moving than others, it is not known how long individual living recommendations remain valid and whether there are particular factors that affect their lifespan.

This study aimed to describe the length of time NICE COVID‐19 living guideline recommendations have remained valid, defined as applicable and relevant to clinical practice without evidence or intelligence indicating the need to update or withdraw recommendations.

## 2 MATERIALS AND METHODS

All NICE guidelines on COVID‐19 published between 1 March 2020 and 22 August 2022 were included. The guidelines were all maintained as living guidelines and therefore eligible for inclusion. The living approach included continuous surveillance for new emerging evidence or intelligence and conducting updates as soon as possible after a trigger for update emerged. A trigger for update was defined as new evidence or intelligence that could impact guideline recommendations. Continuous surveillance included weekly searching for relevant evidence and assessing whether the new evidence would impact current recommendations. In addition, relevant ongoing trials were tracked and checked monthly for publications or data to assess the impact on recommendations. Intelligence was obtained through external enquiries or discussions with committee members to understand the changing context around managing COVID‐19 and this intelligence was considered for impact on the guideline. A framework (see Appendix S2) was developed for the COVID‐19 guidelines, which outlined factors to take into account when considering triggers for update. This framework was based on the team's experience of working on standard NICE guideline surveillance methods. The framework was discussed within the COVID‐19 team whenever a trigger arose, and a decision was made about how to proceed.

Overall, 26 guidelines related to COVID‐19 were included covering service delivery, modifications for other conditions and specific management of COVID‐19 and its complications (see Appendix S1 for the full list). As these were living guidelines, the unit of surveillance and updates was at the recommendation level.

### 2.1 Data extraction

Data were extracted at individual recommendation levels for each guideline into an Excel spreadsheet. Data were extracted by one reviewer and quality was assured by a second reviewer. Disagreements were resolved through discussion with a third reviewer. Data extraction was agreed a priori and recorded in a protocol for reviewers to follow. Key dates were logged including the date of first publication, surveillance trigger date and date of update publishing. Descriptive data were also collated per recommendation including guideline number, guideline title, recommendation number, section number and title, question type, topic area and underpinning evidence base. The 26 guidelines were published in one of two formats, HTML on the NICE website or in MAGICapp. For the HTML guidelines, the recommendation number allocated in the guideline was used as a unique ID, whereas the MAGICapp recommendations were given sequential recommendation numbers manually as an ID (NICE guidelines NG191 and NG188). Each recommendation was categorised into one of the following review types: intervention, diagnosis, prognosis, service delivery, patient experience, aetiology or health inequalities. Intervention recommendations were allocated an additional subcategory of either pharmacological or nonpharmacological. Interventions that were related to therapeutics or medicines for the management of symptoms and disease were allocated to the pharmacological subcategory. Nonpharmacological interventions included management approaches such as respiratory support and rehabilitation. Recommendations were also categorised into one of four types of evidence that were judged to underpin the recommendation including quantitative, qualitative, mixed evidence (defined as both quantitative and qualitative) and committee consensus. The category of committee consensus was used where no references were related to the recommendation. For the other categories, the evidence review used to inform the recommendation was used to judge the evidence type.

Updates were also categorised as minor or major. Major changes to recommendations were defined as those resulting from a new or updated evidence review. Minor changes were defined as those that could be actioned without conducting an evidence review. Minor changes included adding or deleting hyperlinks, amending text for clarity, deleting outdated information, updating information taken from other guidelines and changing supplementary information (such as referring to an updated national policy from an external source). Major changes were categorised as a change in the evidence base including conducting a new meta‐analysis or updating an existing meta‐analysis with new studies or outcomes that resulted in a change or no change in the recommendation wording or intent. Additionally, if a recommendation or whole guideline was withdrawn or incorporated into another guideline, this was also considered a major change.

A decision to update was recorded if a recommendation had undergone surveillance and the decision had been reached to ‘update’. In this scenario, a date of update publishing was added to the recommendation data. The subsequent publication of the updated recommendation was then logged on a new row of the spreadsheet as another data point. The updated recommendation was further assessed for surveillance triggers until withdrawn or censored for survival time.

If a recommendation had undergone surveillance but no update, the decision logged was ‘no update’. If a recommendation had not undergone surveillance, the decision to update was coded as ‘no surveillance’ and censored on 31 August 2022, which was the cut‐off date for the study.

### 2.2 Data analysis

Survival time per recommendation was calculated in weeks from the publication date to the date of update publishing, withdrawal or censored date, in cases where there had not been any surveillance.

#### 2.2.1 Subgroup analyses and rationale

Subgroup analyses were conducted based on distinct review types, encompassing intervention, diagnosis, prognosis, service delivery, patient experience, aetiology and health inequalities. The primary objective of this analysis was to elucidate disparities in recommendation lifespans across diverse review types, offering valuable insights into areas that may necessitate more frequent updates or heightened surveillance.

A further subgroup analysis was carried out within the intervention recommendations category, contrasting pharmacological and nonpharmacological interventions. This evaluation aimed to investigate potential variances in the lifespan of recommendations pertaining to drug therapies and other management approaches, such as respiratory support and rehabilitation.

An additional subgroup analysis compared the lifespans of recommendations based on the underpinning evidence base, which included quantitative, qualitative, mixed evidence and committee consensus. This examination aimed to assess the impact of the type of evidence on the recommendation lifespan and ascertain whether specific types of evidence may contribute to more frequent updates or shorter lifespans.

Lastly, a subgroup analysis compared the lifespan of recommendations when minor and major updates were conducted. This investigation sought to determine whether the type of update (minor changes or significant changes to the evidence base) influenced the lifespan of living guideline recommendations.

### 2.3 Statistics

Stata version 13 software was used to calculate median survival times, interquartile ranges and to plot Kaplan‐Meier survival estimate curves for all data and each subgroup analysis. The median survival time was taken as the time point at which the survival probability dropped to 0.5% or 50%.

Microsoft Excel was used to calculate the duration of time in days between the surveillance trigger dates and the dates of an update publishing or withdrawal of a recommendation. Mean values were then calculated in Excel for all data and each subgroup analysis.

## 3 RESULTS

**Figure 1:**
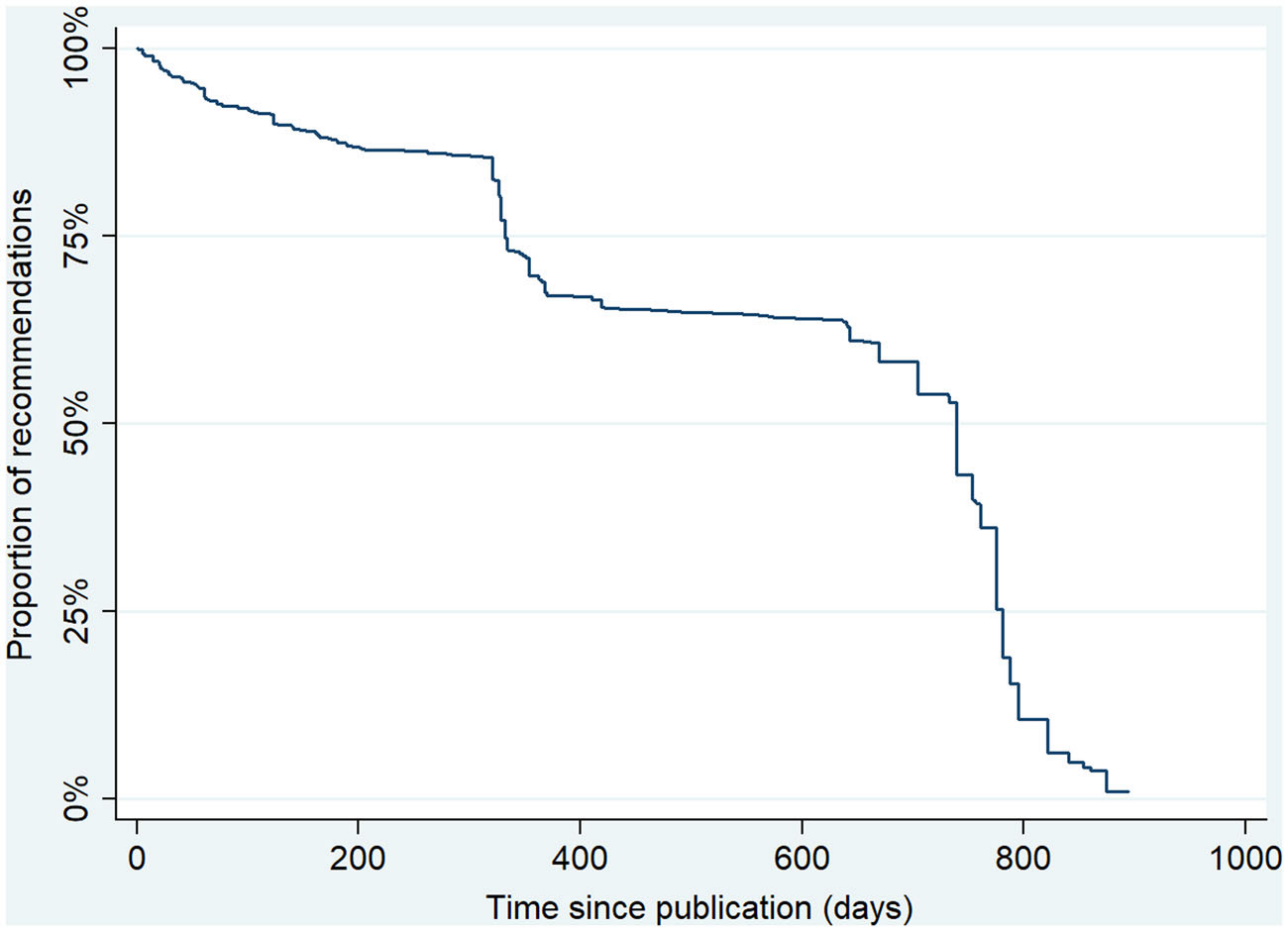
Kaplan–Meier survival curve for all recommendations.

**Figure 2:**
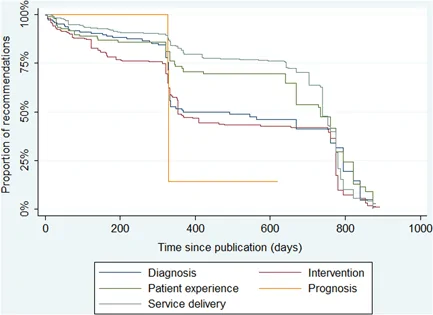
Kaplan–Meier survival curves for the recommendation types.

The results are shown in Table 1. In total, 1182 recommendations were included from 26 COVID‐19 living guidelines. The median survival time of living recommendations was 739 days (interquartile range [IQR]: 332, 781) (see Figure 1). Subgroup analyses indicated that prognostic recommendations had the shortest lifespan compared with service delivery recommendations, which had the longest lifespan (medians of 328 vs. 739 days, IQR: 643, 781, respectively) (see Figure 2). However, only 10 recommendations were categorised as prognostic, so there is considerable uncertainty around this result. Intervention recommendations were found to have the second shortest lifespan at 354 days (IQR: 312, 775) compared to diagnosis (368 days, IQR: 328, 795), patient experience (733 days, IQR: 345, 795) and service delivery (739 days, IQR: 643, 781).

**Table 1:** Results for all categories of analysis.

Category of analysis	Number of recommendations that were published^a^	Total number of recommendation days analysed^b^	Incidence rate of update or withdrawal per day	Mean days from first surveillance trigger to date of update, publishing or withdrawal	Survival time		
					25th percentile	50th percentile (median average)	75th percentile
Total for all recommendations	1182	616,293	0.0015463	29.12	332	739	781
By recommendation type							
Diagnostic	146	58,418	0.0014721	42.41	328	368	795
Interventions: Total	296	112,065	0.0018471	27.78	312	354	775
Interventions: Pharmacological	205	69,584	0.0019401	23.40	161	335	775
Interventions: Nonpharmacological	80	39,748	0.0015850	23.40	354	775	775
Interventions: Pharmacological with nonpharmacological	11	2733	0.0032931	26.22	163	363	368
Patient experience	110	58,542	0.0014690	47.78	345	733	795
Prognostic	10	3464	0.0017321	37.33	326	328	328
Service delivery	620	383,804	0.0014799	24.65	643	739	781
By evidence type							
Consensus	1074	574,791	0.0015275	29.28	334	739	781
Mixed	48	28,587	0.0016441	20.66	463	669	775
Quantitative	60	12,915	0.0021680	38.32	123	321	[c]
By evidence type for the intervention recommendations alone							
Consensus	218	85,390	0.0018620	27.45	321	354	775
Mixed	20	14,276	0.0014010	15.65	775	775	775
Quantitative	58	12,399	0.0022582	38.32	123	205	[c]
By major or minor update							
Major	791	490,329	0.0016132	28.34	363	739	781
Minor	163	30,053	0.0053905	32.92	33	100	328
Major or minor^d^	954	520,382	0.0018313	29.12	328	704	775

^a^ This includes the number of recommendations that were published and updated. In other words, when a recommendation is updated, we consider the update to be a different and new recommendation.

^b^ This may also be called ‘recommendation days at risk’.

^c^ The 75th percentile is not calculable because fewer than 75% of the recommendations in this category were updated or withdrawn by the censor date of 31 August 2022.

^d^ These values are different compared with the total for all recommendations because 228 recommendations were not updated by the censor date of 31 August 22.

**Figure 3:**
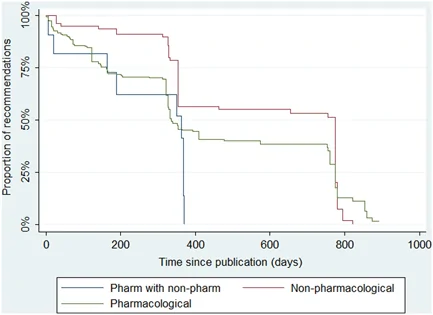
Kaplan–Meier survival curves for intervention recommendation subgroups.

Within the intervention recommendations category, pharmacological recommendations had the shortest survival time compared with nonpharmacological recommendations (medians of 335 days vs. 775 days, respectively) (see Figure 3).

**Figure 4:**
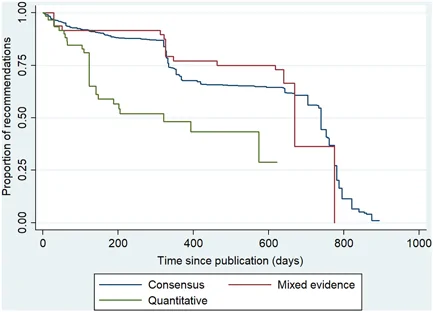
Kaplan–Meier survival curve for recommendation evidence types.

Subgroup analysis comparing the lifespan when considering the underpinning evidence base for the recommendations indicated that quantitative recommendations had the shortest lifespan compared with consensus recommendations (medians of 321 vs. 739 days, respectively) (see Figure 4).

**Figure 5:**
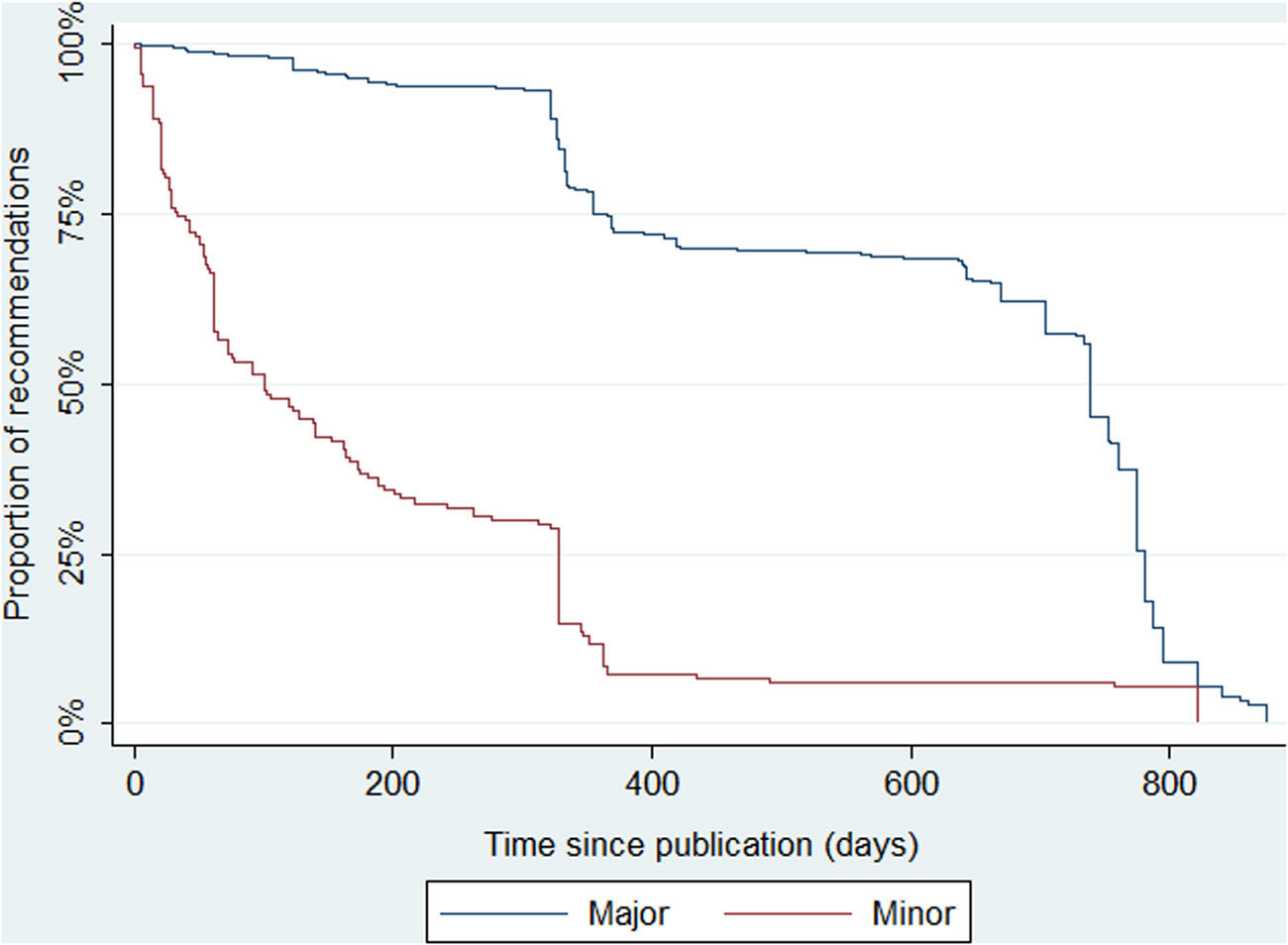
Kaplan–Meier survival curve for major or minor update.

A comparison of update types showed that minor updates have a much shorter lifespan than recommendations with major updates (medians of 100 vs. 739 days, respectively) (see Figure 5).

The mean duration of time from a surveillance decision to publishing an update was 29.12 days.

Mixed evidence had the shortest surveillance trigger date to date of publishing and quantitative evidence had the longest (means of 15.65 vs. 38.32 days, respectively).

## 4 DISCUSSION

To our knowledge, this is the first study investigating the lifespan of living guideline recommendations. The results of the study demonstrated that the lifespan of recommendations differs depending on characteristics such as review type or the underpinning evidence that informed the recommendation. The median survival time of living recommendations was 739 days, which, although shorter than has been reported in previous analyses of full guidelines which have been around 5 years, is possibly longer than anticipated for living guideline recommendations.^6^, ^7^

In this study, it was observed that major updates exhibited a significantly longer survival time in comparison to minor updates (781 vs. 100 days, respectively). This may be attributed to the fact that major updates rely on new evidence emerging that requires an update to an evidence review whereas, in this study, minor updates tended to be editorial in nature and often prompted by user feedback following the publication of a new recommendation. Although the model of continuous surveillance used for these guidelines may have contributed towards a shorter lifespan for the minor updates, the threshold for decision‐making was aligned to standard NICE guideline surveillance, which would help minimise this bias.

Recent studies reporting on methods for living guidelines discuss the importance of prioritising recommendations within a guideline to be living as not all recommendations are expected to require updates at the same pace.^3^, ^8^ Guideline developers may want to consider the recommendations within a living guideline and the factors that could impact how quickly they could become out of date as part of prioritisation for living mode. Subtopic variations within living sections of a guideline may also warrant differing intensities of surveillance, such as by varying the frequency of searching and screening. The value of utilising a flexible responsive approach to surveillance within the living mode enables guideline developers to allocate resources according to the pace of change and expectation of update triggers.^9^

The mean duration of time from a surveillance decision to publishing an update was 29.12 days. This short timeframe is likely to have been driven by a large proportion of major updates than minor updates (791 vs. 163). In subgroup analysis, a shorter mean duration from surveillance to update was noted in recommendations on service delivery (24.65 days) and intervention (27.78 days) compared to recommendations on diagnosis (42.4 days), prognosis (37.3 days) and patient experience (47.7 days). In the total sample, there were 620 recommendations on service delivery and a major update was common for service delivery recommendations as withdrawal of these recommendations. Likewise, there were 296 recommendations on interventions and minor updates were more commonly observed for intervention recommendations. Both service delivery and intervention recommendations constituted a bigger part of our sample and hence may explain the overall shorter duration from surveillance decision to update publishing in the data. Other factors likely contributing to the rapid updates were the infrastructure used to deliver the COVID‐19 living guidelines including reuse of data from other organisations and a rolling expert panel that could be convened when needed. Integrating stages of guideline development, surveillance and updating into a single working model has been reported by other guideline organisations and was shown to enhance the speed of the workflow.^10^

In this study, a number of subgroup analyses were conducted with the aim of understanding what factors might drive how quickly recommendations become out of date and benefit various stakeholders involved in the development and implementation of living guidelines. One subgroup analysis of major and minor updates showed that minor updates have a much shorter lifespan than recommendations with major updates. This difference could be expected given the differing nature of what constituted a major or minor update and the resources required to implement each. For example, major changes to recommendations were defined as those resulting from a new or updated evidence review. Minor changes were defined as those that could be actioned without conducting an evidence review and could typically be actioned more quickly. However, it is possible that major updates had a bigger impact on clinical practice, given that major updates included updates to evidence syntheses that may or may not have resulted in a change in the direction of effect for recommendations.

By understanding the differences in the lifespan of living guideline recommendations across various subgroups, guideline developers can efficiently allocate resources by concentrating on areas that require more frequent updates or surveillance.

A strength of this study is the inclusion of a portfolio of guidelines that had been maintained as living guidelines for 2 years or more. The study is timely and relevant, as it addresses the lifespan of living guidelines in the context of the rapidly changing COVID‐19 pandemic. We performed a comprehensive data extraction approach by extracting data at the individual recommendation level for each guideline, ensuring a detailed analysis. The inclusion of major and minor updates reflected the range of update triggers arising in living guideline surveillance. The study considered different types of recommendations, such as intervention, diagnosis, prognosis and service delivery, which increases its comprehensiveness and generalisability. We performed Kaplan–Meier survival estimate curves and calculated quartiles of survival time, which provides robust and reliable results and performed subgroup analyses to explore the influence of different factors on the lifespan of living guideline recommendations.

Nonetheless, the findings of this study should be considered alongside the following limitations. First, the study lacks external validation of the results, which may affect the generalisability and applicability of the findings to other settings. Additionally, as the recommendations included in this study were all derived from COVID‐19 guidelines, this limits the generalisability of the lifespan of living guideline recommendations, which may differ across disease areas. A unique aspect of COVID‐19 was that this was an area of research in which there was no existing evidence and also where new data were being published rapidly, which is unlikely to apply directly to any other living guideline. The survival times presented in this study are relevant to that particular context. However, it is generally accepted that within the context of a living guideline, recommendations will vary in their lifespan and become out of date at different time points. Therefore, this study does support the concept of prioritising recommendations in more dynamic areas of a guideline to have continuous living status. Guideline developers should consider which recommendations within a living guideline would have the most value in being maintained as fully living to optimise resources.

Due to the volume of guidelines and recommendations, we employed single‐reviewer data extraction. Although data for each guideline were extracted by one reviewer and quality assured by a second reviewer, using multiple reviewers for data extraction could have strengthened the reliability of the data. Linked to this, the categorisation of updates into major or minor changes could be subject to bias, as different people may have different interpretations of what constitutes a major or minor change. We aimed to minimise this by creating definitions for major and minor updates and including these in the study protocol.

Finally, the study does not assess the impact of updated recommendations on clinical practice or patient outcomes, limiting the understanding of the practical implications of the findings.

In summary, this study demonstrates that within living guidelines, some recommendations need to be updated sooner than others and there is value in implementing a flexible responsive approach to surveillance according to the pace of change and expectation of update triggers.

## AUTHOR CONTRIBUTIONS

**Emma McFarlane**: Conceptualization; methodology; investigation; validation; writing—original draft; writing—review and editing; supervision; data curation. **Toby Mercer**: Investigation; methodology; software; writing—review and editing; writing—original draft; formal analysis; conceptualization; data curation. **Steve Sharp**: Conceptualization; investigation; methodology; validation; writing—original draft; writing—review and editing; data curation. **Debra Hunter**: Project administration; resources; writing—original draft; writing—review and editing; methodology. **Kate Kelley**: Resources; supervision; writing—review and editing. **Fiona Glen**: Supervision; resources; writing—review and editing. **Maria Majeed**: Conceptualization; investigation; methodology; writing—original draft; writing—review and editing; data curation; validation.

## CONFLICT OF INTEREST STATEMENT

The authors declare no conflict of interest.

## ETHICS STATEMENT

No ethical approval was needed for this study.

## Supporting information

Supporting information.

Supporting information.

## Data Availability

The data that support the findings of this study are available on request from the corresponding author. The data are not publicly available due to privacy or ethical restrictions.
