# Supplementary material for: Lifespan of COVID‐19 living guideline recommendations: A survival analysis
Source: Clin Public Health Guidel. 2024 Aug 31;1(4):e12012. doi: 10.1002/gin2.12012 (PMC13563357; doi:10.1002/gin2.12012)
Supplement: Supplementary file 1 [file GIN2-1-e12012-s001.docx]

# Appendix 2

**Framework for surveillance triggers used to consider updates for COVID-19 guidelines**

| **Study characteristics** | Study in evidence review is retracted and removing the data changes the direction of effect for critical outcome  Evidence or intelligence highlights conflict with current government policy   - New evidence addresses a research recommendation - Conclusive new evidence is likely to change direction of current recommendation(s) OR if there is no recommendation it could substantiate previously identified evidence which was too weak to form a recommendation - New evidence is a full publication of a pre-print already included in the evidence review AND final data in the full publication have concerning differences from the preprint and this has an important impact on the guideline (e.g. in critical outcomes, changes direction of effect) - New evidence is likely to increase certainty of current recommendation(s) - Potential to add recommendations in new area (such as adding new / relevant subpopulation such as immunocompromised or vaccinated) - Publication status of new evidence is primarily full publications | - Study in evidence review is retracted but removing the data has limited or no impact on direction of effect for critical outcomes - New evidence is a full publication of a pre-print already included in the evidence review AND final data in the full publication has some minor differences - Some of the new evidence is likely to change direction of current recommendation(s) but there are some variable results - Publication status of new evidence is primarily pre-prints | - Publication status of new evidence is exclusively academic in confidence - New evidence reports variable results - New evidence is insufficient (underpowered, anticipated/likely high risk of bias or indirect) and requires further confirmatory evidence (i.e. potential future impact) |
| --- | --- | --- | --- |
| **Trial results** | - Potential signal for harm - Likely change in direction for one or more critical outcomes - New evidence provides data on outcomes in the PICO that have not been considered | - New evidence provides data on outcomes in the PICO that have not been considered - Likely change in direction for at least one critical outcome | - Informing important outcomes only - If data relevant to critical outcomes, it is likely to be supportive (i.e. not changing direction of effect) |
| **Current context** | - New evidence is from a platform trial / study set within the UK context so directly relevant to population covered by the guideline - Request to reconsider from panel members | - New evidence is from a platform trial / study set within the UK context so directly relevant to population covered by the guideline - There have been some queries on the area coming through external communications | - New evidence is from studies conducted outside of the UK or in populations that may have less applicability to current context (e.g. standard of care provided in studies is different to current context) |
| **Stability of current recommendation(s) – (topic area is contentious/subject of uncertainty)** | - Current recommendation(s) have potential to change as currently ‘only in research, or conditional recommendations’ - Area has been updated previously either once or several times | - Current recommendation(s) have potential to change as currently ‘only in research, conditional or consensus recommendations’ - No previous update or one previous update | - Current recommendation(s) are strong ‘to use or do not use’ - Current recommendations are overarching and reflect established current practice - No previous update |
| Action | Plan for immediate update | Plan for update when capacity allows | Keep monitoring until strong trigger occurs |
