# Supplementary material for: Lifespan of COVID‐19 living guideline recommendations: A survival analysis
Source: Clin Public Health Guidel. 2024 Aug 31;1(4):e12012. doi: 10.1002/gin2.12012 (PMC13563357; doi:10.1002/gin2.12012)
Supplement: Supplementary file 2 [file GIN2-1-e12012-s002.docx]

# Appendix 1

| Guideline No. | Guideline title |
| --- | --- |
| NG159 | Critical care in adults |
| NG160 | Dialysis service delivery |
| NG161 | Delivery of systemic anticancer treatments |
| NG162 | Delivery of radiotherapy |
| NG163 | Managing symptoms (including at the end of life) in the community |
| NG164 | Haematopoietic stem cell transplantation |
| NG165 | Managing suspected or confirmed pneumonia in adults in the community |
| NG166 | Severe asthma |
| NG167 | Rheumatological autoimmune, inflammatory and metabolic bone disorders |
| NG168 | Community-based care of patients with chronic obstructive pulmonary disease |
| NG169 | Dermatological conditions treated with drugs affecting the immune response |
| NG170 | Cystic fibrosis |
| NG171 | Acute myocardial injury |
| NG172 | Gastrointestinal and liver conditions treated with drugs affecting the immune response |
| NG173 | Antibiotics for pneumonia in adults in hospital |
| NG174 | Children and young people who are immunocompromised |
| NG175 | Acute kidney injury in hospital |
| NG176 | Chronic kidney disease |
| NG177 | Interstitial lung disease |
| NG178 | Renal transplantation |
| NG179 | Arranging planned care in hospitals and diagnostic services |
| NG186 | Reducing the risk of venous thromboembolism in over 16s with COVID-19 |
| NG187 | Vitamin D |
| NG188 | Managing the long-term effects of COVID-19 |
| NG191 | Managing COVID-19 |
| NG200 | Vaccine-induced immune thrombocytopenia and thrombosis (VITT) |
